# Supplementary figures and images for: Lysis of arterial thrombi by perfusion of N,N’-Diacetyl-L-cystine (DiNAC)
Source: PLoS One. 2021 Feb 25;16(2):e0247496. doi: 10.1371/journal.pone.0247496 (PMC7906380; doi:10.1371/journal.pone.0247496)

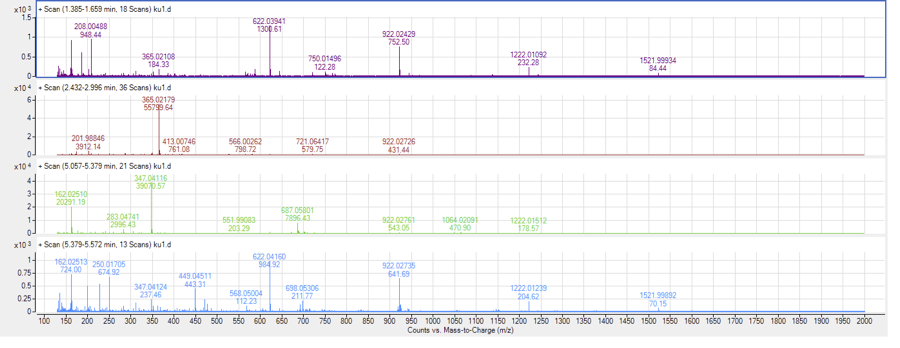

Supplement: S1 Fig — (TIF) [file pone.0247496.s001.tif]

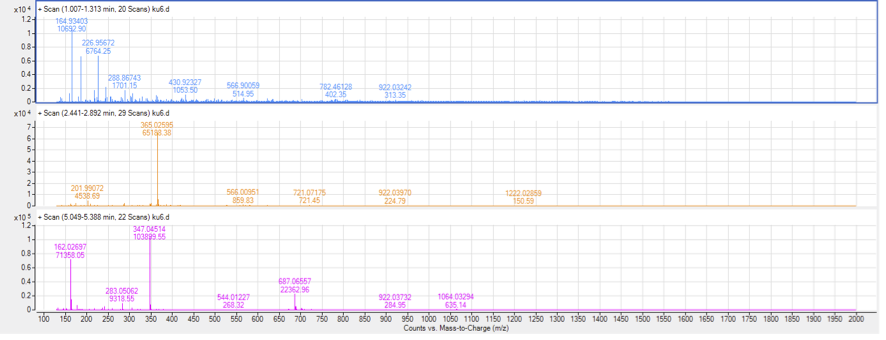

Supplement: S2 Fig — (TIF) [file pone.0247496.s002.tif]
