# Supplementary material for: Lysis of arterial thrombi by perfusion of N,N’-Diacetyl-L-cystine (DiNAC)
Source: PLoS One. 2021 Feb 25;16(2):e0247496. doi: 10.1371/journal.pone.0247496 (PMC7906380; doi:10.1371/journal.pone.0247496)
Supplement: Attachment — Submitted filename: Kim et al. Lysis of arterial thrombi by perfusion of DiNAC.docx [file pone.0247496.s010.docx]

This article by Kim et al. demonstrated findings that support the thrombolytic capacity of DiNAC towards arterial thrombi. The authors should be commended for their demonstrated ingenuity and scientific skills towards investigating DiNAC alongside several other thrombolytic agents, in the context of evaluating their thrombolytic capacity of white clots and red clots. It is clear that these findings will continue to support research into fibrinolysis and its role in therapeutic thrombolysis. However, there are some major and minor points for consideration. These considerations should be reflected in the manuscript prior to its acceptance for publication. Please see these points below.

Major comments

1. Given that porcine blood was utilised across all experiments, but all pharmacological agents (except NAC and DiNAC) are matched for human use, there is the possibility that the results observed are attributed to the species differences between blood and pharmacological agents. Therefore, consider performing the same experiment with human blood and the same pharmacological agents or maintaining porcine blood use but with porcine-derived pharmacological agents. Although these differences are expected to be minimal (line 362), the referenced paper does not elaborate as to how these differences are expected to be negligible in thrombolysis (it only refers to thrombus formation and not its similarities in the context of thrombolysis).
2. Please consider using plasmin (with correct species matching between pharmacological agent and blood) as a positive control, particularly for the experiment under flow conditions.

3. Please elaborate as to how occlusive white thrombi was determined to be 90% (line 169).
4. Please further define the statistical analysis performed for each figure. The data analysis sub-heading in the materials and methods describes the use of ANOVA (line 155). However, please include the specific ANOVA test that was used for each figure (detailed in each respective figure caption). Please include the post hoc analysis performed as well.

Minor comments

1. Apart from its adjuvant role in thrombolysis, Abciximab is not considered a classically defined thrombolytic agent (it is considered an anti-thrombotic). Therefore, consider rephrasing the wording throughout the manuscript so that abciximab is not implied as a thrombolytic agent.
2. For future reference, to test clot lysis further there is a simple and high-throughput assay referred to as the ‘Halo’ assay (Bonnard et al. 2017).
