# Supplementary material for: Lysis of arterial thrombi by perfusion of N,N’-Diacetyl-L-cystine (DiNAC)
Source: PLoS One. 2021 Feb 25;16(2):e0247496. doi: 10.1371/journal.pone.0247496 (PMC7906380; doi:10.1371/journal.pone.0247496)
Supplement: Attachment — Submitted filename: response4.docx [file pone.0247496.s011.docx]

Response to reviewer

Reviewer 1

Major comments

1. Given that porcine blood was utilised across all experiments, but all pharmacological agents (except NAC and DiNAC) are matched for human use, there is the possibility that the results observed are attributed to the species differences between blood and pharmacological agents. Therefore, consider performing the same experiment with human blood and the same pharmacological agents or maintaining porcine blood use but with porcine-derived pharmacological agents. Although these differences are expected to be minimal (line 362), the referenced paper does not elaborate as to how these differences are expected to be negligible in thrombolysis (it only refers to thrombus formation and not its similarities in the context of thrombolysis).

🡪 Thank you for your comment. We found our tPA worked for red clot lysis at comparable rates when compared to the other assays in literature, although ADAMTS-13 and/or abciximab may not work well with porcine blood. We have added text to the manuscript to clarify this limitation (line 371). Our study focused on the efficacy of DiNAC, as this is the first report to find thrombolytic efficacy of DiNAC in our best knowledge. We are planning to conduct human blood experiments and in vivo mice studies in the future to verify efficacy of each agent under different species.

2. Please consider using plasmin (with correct species matching between pharmacological agent and blood) as a positive control, particularly for the experiment under flow conditions.

🡪 Yes, plasmin could be very useful as a positive control if it lyses white clot effectively, and we have looked into this approach. Unfortunately, plasmin is prohibitively expensive with the capillary tube assay utilized for these studies. For n=5, it would cost ~$120,000 for max solubility (1mg/ml, Sigma Aldrich, p1867). Plasmin could be applicable for smaller assays, such as microfluidics and in vivo mice models, which we plan in the future.

3. Please elaborate as to how occlusive white thrombi was determined to be 90% (line 169).

🡪 Thank you for your comment. Blood flow was paused when the upstream pressure reached 30 mmHg, thus assuming Poiseuille flow and thrombi growth from the glass wall, white thrombi is more than 80% occlusive by area. The following sentence is added to the text.

“Assuming a Poiseuille flow and thrombi growth from the glass wall, 30 mmHg pressure gradient can be resulted from > 80% occlusive white thrombi by area.”

4. Please further define the statistical analysis performed for each figure. The data analysis sub-heading in the materials and methods describes the use of ANOVA (line 155). However, please include the specific ANOVA test that was used for each figure (detailed in each respective figure caption). Please include the post hoc analysis performed as well.

🡪 Thank you for your comment. One-way ANOVA was used with Tukey’s post hoc test for multiple group comparison. This specific test has been added to the methods section.

Minor comments

1. Apart from its adjuvant role in thrombolysis, Abciximab is not considered a classically defined thrombolytic agent (it is considered an anti-thrombotic). Therefore, consider rephrasing the wording throughout the manuscript so that abciximab is not implied as a thrombolytic agent.

🡪 Thank you for your comment. Yes, in general, abciximab is used as an anti-thrombotic agent. We added text in the manuscript to clarify this (line 79).

2. For future reference, to test clot lysis further there is a simple and high-throughput assay referred to as the ‘Halo’ assay (Bonnard et al. 2017).

🡪 Thank you for your comment. We will consider using ‘Halo’ assay for future studies.

Reviewer 2

The study is overall very interesting. The efficacy of thrombolysis changing according to blood clot composition is an important question for which clear answer is difficult to get. In vitro study comparing different clot type and different thrombolytic such as this study is therefore very important for the field. However, there are some important lack of data to support some claims, in particular regarding the difference of efficacy between diNAC and NAC. I recommend major revision and would be grandly appreciated if the authors could answer several points:

1) The results section start with
« Initial perfusion with NAC showed promising lytic results. However, replicate perfusion experiments with solutions made with NAC purchased from a variety of vendors yielded nconsistent results with minimal lysis. To further investigate this contradiction, we analyzed the initial solutions with high thrombolytic efficacy using mass spectrometry and found a large peak of DiNAC (data not shown) »

The author should provide the details of these experiments, if several batch of NAC have been tested, the details of the batches should be provided, along with their respective efficacy and the analysis that were performed, including mass spectrometry analysis.

Alternatively, the authors should remove this paragraph from the results section and present in a first figure a mass spectrometry analysis of the NAC (Thermo Fisher Scientific, Waltham, MA) and the DiNAC (Cayman Chemical, Ann Arbor, MI).

Alternative methods showing the difference between NAC and DiNAC are also acceptable.

🡪 Thank you for your comment. We referenced initial perfusion with NAC and added mass spectrometry data as a Supplemental figure 1 and 2.

Supp Fig 1. Mass spectrometry data of DiNAC solution.

Supp Fig 2. Mass spectrometry data of NAC solution.

2) The supplemental videos are much appreciated. However, one video from each group should be provided.

🡪 We have added video for each group (PBS, NAC, tPA, ADAMTS13, and Abciximab) as supplemental material.

3) Additional experiments showing a difference of activity from NAC and DiNAC would be appreciated. If DiNAC has a reduction potential higher than NAC, it could be measured in vitro with basic molecular biology methods. It could be tested on soluble vWF maybe as well ? Analysed with SDS-PAGE with non denaturing condition ?
Alternatively, the authors should state what they have tested and explain why no difference could be seen between NAC and DiNAC.

🡪 Thank you for your comment. We do think the mechanism of DiNAC is an interesting topic, we focused on the efficacy of DiNAC and leave the mechanistic part as a future study. The suggested study is not trivial as our first tests in this direction have not yielded differential results. We have thus chosen to publish these initial findings so that others can help seek out a good explanation. However, we have added a comment about a potential mechanism in discussion session.

4) The discussion should provide hypothesis on why DiNAC is more potent than NAC in vitro.

🡪 Thank you for your comment. We edited following text why DiNAC could work better than NAC.

“The mechanism of thrombolysis of white clot by DiNAC is an interesting topic as DiNAC does not contain a free thiol, which is essential for NAC to cleave VWF multimers [29] or any other blood components that has sulfide bonds which could limit delivery of NAC to VWF. Instead, DiNAC has a sulfide bond that can be reversed to two thiols [54]. Thus, DiNAC may be delivered into the clot and converted to two NACs before reacting with VWF, or there could be a new reaction that induces switching between two disulfide bonds. We leave this topic for a future study.”

Minor comments :

There several spelling mistakes that should be corrected.

🡪 Thank you, we have double checked our grammar and spelling and made changes accordingly.
